# Perceived parental encouragement on self-efficacy and resilience of Chinese college students

**DOI:** 10.3389/fpsyg.2026.1700484

**Published:** 2026-04-10

**Authors:** Jiwei Wu, Holly Swearingen, Changming Duan

**Affiliations:** Department of Educational Psychology, University of Kansas, Lawrence, KS, United States

**Keywords:** Chinese college students, family environment, perceived parental encouragement, resilience, self-efficacy, well-being

## Abstract

**Introduction:**

This study examined the role of perceived parental encouragement (PPE) in self-efficacy and resilience among Mainland Chinese college students and whether family environmental factors moderated these associations.

**Method:**

Using a cross-sectional correlational design, 185 participants from 48 universities across 18 cities in China completed measures of PPE, self-efficacy, resilience, and family environment, including Conflict, Cohesion, and Expressiveness. Correlational, regression, and moderation analyses were conducted.

**Results:**

Results indicated that PPE was positively associated with both self-efficacy and resilience and significantly predicted both outcomes. Family Conflict significantly moderated the association between PPE and resilience. Higher Family Conflict weakened the positive relationship between PPE and resilience. Family Cohesion and Expressiveness did not significantly moderate associations between PPE and either outcome. Additional differences were found across gender, only-child status, and city tier.

**Conclusion:**

These findings suggest that PPE may serve as an important family-related resource for psychological well-being among Chinese college students, but its positive association with resilience may be attenuated in high-conflict family environments. The findings highlight the importance of considering both parental support and harmony in understanding psychological well-being within the Chinese cultural context.

## Introduction

Encouragement, as a psychological concept introduced by Alfred Adler (1937), has been viewed as both an action and a way of being that aims at giving an individual a sense of respect and confidence (Dreikurs, 1964). It is also recognized as a form of social support, involving providing positive affirmations, motivation, and belief in an individual’s abilities (Wong, 2015). Emerging evidence shows that parental encouragement plays a critical role in adolescents’ wellbeing, such as autonomy (Fousiani et al., 2014), peer resistance (Wang et al., 2024), sociocultural adjustment (Benitez-DeVilbiss, 2023), academic performance (Agrawal and Pande, 1997), self-efficacy (Bandura, 1977), and resilience (Levit, 1996). Accompanied by parental warmth and involvement that provides a supportive backdrop for development, parental encouragement directly addresses an adolescent’s belief in their ability to overcome challenges (Kiel et al., 2016) and helps foster a positive self-concept and a proactive attitude toward life’s obstacles (Zhou et al., 2008). Similarly, Rueger et al. (2010) found that parental support can motivate children to engage in learning actively.

In general, self-efficacy and resilience are key factors in adolescents’ wellbeing and adaptive functioning (Andretta and McKay, 2020). Self-efficacy refers to beliefs about one’s capability to organize and execute actions required to attain desired outcomes (Bandura, 1977). Research has shown that adolescents who perceive greater parental encouragement are more likely to develop robust self-efficacy (Masten and Reed, 2002). Furthermore, high self-efficacy improves adolescents’ performance across various areas, including academics, professional conduct, and healthy behaviors (Stajkovic and Luthans, 1998). Resilience, defined as the ability to recover and grow positively from stress, adversity, or significant challenges, is a fundamental aspect of healthy development (Liu et al., 2012). Empirical evidence has shown that a lack of parental support is linked to increased psychological distress and emotional difficulties (Helsen et al., 2000).

Encouragement can be conveyed through direct verbal affirmation (e.g., praise), non-verbal gestures showing approval (e.g., a nod or a pat on the back), and indirect communication (e.g., showing interest or excitement in something, the adolescent initiated; Benitez-DeVilbiss, 2023). However, the effectiveness of expressed encouragement depends on the extent to which the adolescent internalizes and believes in the intended message. When children first develop their sense of self and learn to navigate challenges, parental messages play a vital role in their mental health and academic success. Encouragement must be infused into the parenting process to effectively establish a supportive parent–child relationship that provides a safe space for children to explore their capabilities and develop the confidence needed to face adversity (Morris et al., 2007; Silk et al., 2013).

Self-efficacy and resilience are shaped not only by individual capacities but also by adolescents’ perceptions of their social environments, particularly the degree to which parents communicate confidence, support initiative, and endorse adolescents’ emerging autonomy. Recent work suggests that autonomy-supportive parenting practices are increasingly evident in contemporary Chinese families as part of emotionally supportive and developmentally facilitative approaches to child-rearing (Bao et al., 2023).

Family environment is a key factor in children’s psychological health development (Levit, 1996). When a positive family environment (high Expressiveness, high Cohesion, high Organization, low Control, and low Conflict) was present, children’s self-esteem and sense of mastery were optimized (Hamid et al., 2003). Conversely, when Cohesion is lacking, Conflict is high, or Marital Satisfaction is low in the home, children may experience low family harmony and higher depressive symptoms (Yu et al., 2015). Consistent with attachment theory (Bowlby, 1971) and Ecological Systems Theory (EST; Bronfenbrenner, 1979), the family exerts the most direct and enduring influence on development.

There has been limited research demonstrating the efficacy of parental encouragement on children’s development (e.g., emotional regulation, resilience, and academic performance). Still, little attention has been given to understanding the role of encouragement in Chinese adolescents’ mental health (Morris et al., 2007). In China, adolescents are growing up in a contemporary culture that blends Eastern and Western traditions and norms, while undergoing rapid changes due to modernization and economic development (Zhang and Thomas, 1994). They face increasing high pressure to navigate a complex blend of high academic expectations, traditional familial obligations, and societal norms, which makes the promotion of psychological wellness a personal pursuit and a cultural necessity (Shek and Sun, 2013). Parental encouragement and support were found to be associated with children’s feelings of being challenged and supported, leading to higher levels of psychological wellbeing and a greater capacity to manage stress and adversity (Shengyao et al., 2024). Thus, it is especially important that effective parenting integrates positive practices, such as encouraging positive behaviors. To encourage research and effective practice in Chinese parenting, the current study was designed to examine perceived parental encouragement (PPE) and its role in adolescents’ wellness as indicated by self-efficacy and resilience.

### Chinese cultural context

Grounded in Confucian values, traditional Chinese parenting practices are often parent-centered, emphasizing control, obedience, and academic excellence as markers of familial honor and success (Shek and Sun, 2013). This parenting style aims to cultivate seven Confucian developmental goals in children: knowledge, social norms, modesty, shame, self-restraint, filial piety, and harmonious relationships (Luo et al., 2013). From a Western psychological perspective, the emphasis on respect for authority and conformity to social norms may compromise individuals’ autonomy and Self-Expressiveness, which may result in lower self-efficacy, an undesirable trait associated with negative behavioral consequences (Ho and Hau, 2010). However, Chinese scholars have pointed out that Chinese parenting needs to be understood in context. For instance, the common method, *guan* (管), which oversees or disciplines in English, in fact, carries a positive connotation of “not only to govern but to teach and educate,” for example, taking care of their needs, providing financial support, giving them gifts, and making them food (Liu et al., 2022).

In contemporary times, Chinese parents are increasingly open to integrating emotionally supportive and encouraging practices into parenting. Recent studies show that while traditional practices focus on discipline and achievement, some parents have incorporated various methods of encouragement into parenting, which resulted in children’s psychological flexibility (self-efficacy and lifestyle choices) and emotional intelligence (identity exploration, self-confidence, and autonomy/independence; Bao et al., 2023; Ma et al., 2022). Supportive parenting styles have also been linked to academic resilience, with self-efficacy as one potential mediator (Shengyao et al., 2024). Together, these contemporary findings suggest that parental encouragement may operate as a family-based resource that supports adolescents’ self-efficacy and resilience. This evolution reflects Chinese parents’ commitments to their children’s mental health and emotional wellbeing.

*Gender differences*. It is also critical to recognize that interpretations of parental encouragement and emotional support may vary across adolescents. In particular, gendered socialization processes and culturally embedded expectations regarding boys’ and girls’ academic performance, emotional expression, and family roles may influence both how parents provide support and how youth perceive that support. Recent evidence from Chinese adolescent samples indicates that family functioning and resilience-related processes can vary by gender (Zhang et al., 2024). When linking parenting experiences to developmental outcomes, family-based mechanisms (e.g., psychologically controlling practices and the quality of the parent–child relationships) may operate differently for boys (externalizing factors, e.g., independence and competitiveness) and girls (internalizing factors, e.g., prosocial relationships), showing the importance of examining gender as a contextual factor (Geng and He, 2021; Wang et al., 2024). The current study reviews gender differences in parental support and considers how gender may condition associations between perceived parental encouragement and adolescents’ self-efficacy and resilience.

*Only-child status*. China’s family planning legal policies have shifted over time (from the one-child policy era to policy allowing two children and now the current three children policy), and many adolescents and college students have therefore grown up in either only-child or multi-child families. Following policy changes and the increasing presence of multi-child families, researchers have emphasized that sibling structure and within-family processes (e.g., parental differential treatment) may meaningfully shape children’s perceptions of support and relational security. Research indicates that in Chinese two-child families, parents may treat siblings differently, and this variation in treatment is linked to the quality of sibling relationships. These patterns imply that children’s sense of fairness and the overall emotional climate at home can shape their psychosocial development and the support they receive (Ye et al., 2025). Moreover, research continues to treat only-child status as a meaningful contextual factor in Chinese adolescents’ development, with some evidence suggesting that associations between only-child status and adjustment may vary by demographic and ecological conditions (e.g., gender and school location; Jia et al., 2022). Large-scale contemporary research also suggests that growing up without siblings may be associated with measurable differences in adult brain and behavioral outcomes, reinforcing the importance of attending to only-child vs. multi-child contexts when interpreting family support in modern China (Tang et al., 2025).

*City tier*. In China, researchers often use city-tier classification to characterize participants’ broader living environments, as these tiers reflect regional and urbanization differences that may influence adolescents’ developmental opportunities and the everyday stressors they encounter. Recent Chinese studies examining psychological wellbeing, such as self-efficacy and resilience, have reported city tier as part of the demographic profile and/or included it as a covariate in models of psychological outcomes (Zhang et al., 2025; Li et al., 2025). The present study, therefore, includes city tier as a demographic factor to contextualize adolescents’ experiences and explores whether associations among parental encouragement, self-efficacy, and resilience vary across broader residential contexts. While recognizing differences across city tiers, researchers should interpret results descriptively rather than as evidence of specific causal mechanisms.

### The present study

This study investigated the role of perceived parental encouragement (PPE) in self-efficacy and resilience among Chinese college students. Additionally, the study examined family environmental factors (Family Cohesion, Family Conflict, and Family Expressiveness) as moderators of the relationships between encouragement and self-efficacy and between encouragement and resilience. We hypothesized that PPE would be positively associated with self-efficacy and resilience, respectively. We further hypothesized that Family Expressiveness, Family Conflict, and Family Cohesion would moderate these two relationships. Specifically, we hypothesized that higher Family Expressiveness and Family Cohesion would be associated with a stronger relationship between PPE and self-efficacy. In contrast, the lower Family Conflict would be associated with a stronger relationship between PPE and resilience. The study was conducted using the Chinese language, and the results were translated into English for the purpose of this manuscript.

## Methods

### Procedure

This study employed a cross-sectional, correlational design approved by the University IRB (STUDY00150964). Participant recruitment was conducted through online postings on social media, WeChat. Volunteers interested in participating may click the link provided to access Qualtrics. The first page of the survey presented an IRB-approved information statement that described the study’s purpose, the voluntary nature of participation, confidentiality of responses, and the researcher’s contact information. The information statement explicitly stated that clicking “Next” served as electronic informed consent, indicating that the participant had read and understood the study information and their rights as a participant, and had voluntarily agreed to participate. Thus, consent was obtained through an active action (clicking “Next”) to proceed beyond the information statement; individuals who did not wish to participate could exit the survey at that point without penalty. Participants were then prompted to complete the Parental Autonomy Support Scale (PASS), General Self-Efficacy Scale – Chinese Version (GSES-CV), 10-Item Connor-Davidson Resilience Scale – Chinese Version (CD-RISC-10-CV), Family Environment Scale – Chinese Short Version (FES-C-SV), and the demographics form. At the end, participants were thanked for their voluntary participation and directed to exit the survey. Materials and analysis code for this study are available by emailing the corresponding author. This study was not preregistered.

### Participants

The participants were recruited from 48 universities in 18 different cities in China. Initially, 246 students filled out the survey. Among them, 61 cases were excluded due to missing data, and two cases were retained with all survey scale items completed except demographic items, leaving 185 participants’ (*N* = 185, 100 men, 79 women, and 6 self-identified as non-binary) data for analysis. The age range was 18–25 years old (*M* = 20.59, standard deviation [SD] = 1.519). Ethnicity includes Han (*n* = 171), Yi (*n* = 3), Tujia (*n* = 2), Man (*n* = 2), and Mongolian, Uyghur, Yao, and Bai ethnicity groups (*n* = 1), respectively, and giving no answer (*n* = 3). The sample includes Freshmen (*n* = 17), Sophomores (*n* = 70), Juniors (*n* = 78), and Seniors (*n* = 20). Participants are either an only-child (*n* = 77) or have one or more siblings (*n* = 107); one participant did not report their answer (*n* = 1). Participants reported their parental marital status, including married (*n* = 163), divorced (*n* = 14), and widowed (*n* = 6), and two participants did not report their parental marital status (*n* = 2). Participants reported living with both parents (*n* = 168), with one parent (*n* = 12), or with other caregivers (*n* = 3), and two participants did not report their living situation (*n* = 2; see Table 1).

**Table 1:** Demographics.

	*n*	%
Gender		
Female	79	43
Male	100	54
Non-binary	6	3
Ethnicity		
Han	171	94
Yi	3	2
Tujia	2	1
Man	2	1
Other	1	>1
No response	3	2
Class year		
Freshman	17	9
Sophomore	70	38
Juniors	78	42
Seniors	20	11
Only-child status		
Only child	77	42
Multi-child	107	58
Parental marital status		
Married	163	89
Divorced	14	8
Widowed	6	3
Living situation		
Both parents	168	92
One parent	12	6.5
Other caregiver	3	1.5
City tier		
Tier 1	89	49
Tier 2	18	10
Tier 3	10	5
Tier 4	66	36

Tier 1 is most developed (Beijing, Shanghai, etc.); Tier 2 is provincial capitals, fast-growing cities (Nanjing, etc.); Tier 3 is medium-sized cities, with urbanization (Zhuhai, Wenzhou, etc.); Tier 4 is smaller cities and less-developed infrastructure (Shijiazhuang, etc.).

### Measures

*Parental Autonomy Support Scale (PASS)*. In this study, perceived parental encouragement (PPE) is defined as adolescents’ subjective perceptions of parental behaviors that convey confidence, validation, positive affirmation, and support for autonomy. PPE is measured using the Parental Autonomy Support Scale (PASS; Wang et al., 2007), which includes items reflecting encouragement-related parenting behaviors such as promoting independence, supporting decision-making, and acknowledging adolescents’ perspectives. This study, therefore, adopts the term PPE to emphasize adolescents’ experiences and relational factors, rather than framing them solely in terms of parental autonomy support as a parenting orientation. This framing construct allows us to focus analytically on encouragement as adolescents receive and interpret it, while remaining grounded in the theoretical foundations and empirical validity of the PASS. At the same time, we acknowledge that encouragement is a broader construct, and the PASS captures a theoretically central dimension of encouragement rather than the full range of encouraging parenting practices.

The PASS developed by Wang et al. (2007) was used to measure perceived parental encouragement. The PASS contains 12 items that assess behaviors typically categorized as parental encouragement. For example, items such as “My parents encourage me to be independent” and “My parents allow me to make my own decisions.” PASS uses a 5-point Likert scale from 1 (*strongly disagree*) to 5 (*strongly agree*), with a total score of 12–60. A low score indicates low parental encouragement, while a high score indicates high parental encouragement. PASS has good reliability and validity across diverse cultural contexts, including Chinese families. In Wang et al. (2007), confirmatory factor analysis revealed Comparative Fit Index (CFI) > 0.94, Tucker–Lewis Index (TLI) > 0.90, and Root Mean Square Error of Approximation (RMSEA) < 0.06, indicating good construct validity. In the current study, Cronbach’s *α* was 0.90, showing strong reliability.

*Simplified Chinese version of the General Self-Efficacy Scale (GSES-CV).* GSE-CV is a 10-item self-report measure of self-efficacy. It employs a 4-point Likert scale ranging from 1 (*not at all true*) to 4 (*exactly true*). Sample items include “I can always manage to solve difficult problems if I try hard enough” and “I am confident that I could deal efficiently with unexpected events.” The total scores ranged from 10 to 40, with higher scores signifying greater self-efficacy (Schwarzer and Jerusalem, 1995). Zeng et al. (2022) reported a good internal consistency (*α* = 0.91). In our study, Cronbach’s *α* value was 0.89, which was deemed acceptable.

*Ten-Item Connor-Davidson Resilience Scale-Chinese Version (CD-RISC-10-CV)*. Resilience was measured using the CD-RISC-10-CV. Specifically, CD-RISC-10-CV assesses an individual’s capacity to cope with adversity and stress (Ye et al., 2017). The CD-RISC-10-CV is a shorter version of the original 25-item scale, focusing on the core aspects of resilience (Connor and Davidson, 2003). Participants respond to each item on a 5-point scale ranging from 0 (*not true at all*) to 4 (*true nearly all the time*). Example items include “I am able to adapt when changes occur” and “I tend to bounce back after illness or hardship.” According to Cheng et al. (2020), CD-RISC-10-CV showed good reliability in the non-clinical sample (*α* = 0.92). Cronbach’s *α* was 0.91 and 0.88 for men and women, respectively. In our study, Cronbach’s *α* value was 0.86, showing acceptable reliability.

*Family Environment Scale Chinese Short Version (FES-C-SV).* We used the FES-C-SV to assess the family environment. The scale comprises three subscales: Cohesion, Expressiveness, and Conflict. Cohesion assesses family helpfulness, togetherness, and functioning as a unit; Expressiveness evaluates the openness of verbal expression and the emotionality within a family; and Conflict measures the hostility and aggression present within a family (Moos et al., 1974). Those three subscales are considered relevant in Chinese family contexts. Xue et al. (2014) showed good reliability for Cohesion (*α* = 0.90), Expressiveness (*α* = 0.70), and Conflict (*α* = 0.92). In the present study, the reliability coefficients for Cohesion (*α* = 0.77), Expressiveness (*α* = 0.56), and Conflict (*α* = 0.74) were deemed acceptable.

*Demographics Questionnaire*. To provide context for the collected data on the major variables, we collected participants’ demographics (gender, age, class year, and parental marital status). Notably, we also included the following (see Table 1):

a) City tier: China’s city tier system is an informal classification that ranks cities based on their economic development, infrastructure, population size, and administrative significance. The prestige and resources of a city can significantly influence residents’ experiences. We categorize our data into four city tier groups. The first city tier group are the most developed and influential cities with strong economies, advanced infrastructure, and high living standards, such as Beijing and Shanghai; the second group are important provincial capitals and regional centers with fast-growing economies and improving infrastructure, such as Nanjing; the third group are medium-sized cities with developing industries and expanding urbanization, such as Zhuhai, Wenzhou; and the fourth group are smaller cities and county-level urban centers with lower Gross Domestic Product (GDP), slower growth, and less-developed infrastructure such as Shijiazhuang.
b) Only-child status: Due to China’s Only-child policy (1980–2016) that restricted each married couple to give no more than one birth, the majority of the generation born after 1980 are the only child in their family. However, there were individuals with siblings due to the policy exceptions made for ethnic minorities, rural populations, remarried couples, and other special circumstances. Thus, only-child status is a relevant demographic variable to consider.

## Results

### Descriptive statistics and preliminary analyses

We first conducted a normality test to examine the distribution of PPE, self-efficacy, and resilience. PPE and self-efficacy are not normally distributed; therefore, we used a robust statistical analysis of bootstrapping to ensure validation of the inference of the data. PPE was coded into three levels (Low, Medium, and High) using quantile-based grouping for further analysis. Results of the descriptive statistical analysis for the major study variables, including PPE, self-efficacy, resilience, family environment factors (Family Expressiveness, Family Cohesion, and Family Conflict), and demographics, are presented in Table 2. The correlations between PPE and both self-efficacy (*r* = 0.27, *p* < 0.001) and resilience (*r* = 0.38, *p* < 0.001) were significant, indicating that higher levels of PPE were associated with greater self-efficacy and resilience. Additionally, correlations between both Family Cohesion (*r* = 0.31, *p* < 0.01) and Family Expressiveness (*r* = 0.35, *p* < 0.01) with resilience were significant. Correlations between Family Cohesion (*r* = 0.22, *p* < 0.01) and Family Expressiveness (*r* = 0.22, *p* < 0.01) with self-efficacy were significant. PPE was significantly correlated with Family Cohesion (*r* = 0.49, *p* < 0.01) and Family Expressiveness (*r* = 0.42, *p* < 0.01).

**Table 2:** Pearson correlations among variables.

Correlation												
Variable	*N*	Mean	SD	1	2	3	4	5	6	7	8	9
1. Parental autonomy support	185	43.38	7.85	——								
2. Resilience	185	35.00	6.18	0.377^**^	——							
3. Self-efficacy	185	25.95	5.41	0.266^**^	0.683^**^	——						
4. Conflict	185	3.57	2.37	−0.445^**^	−0.132	−0.119	——					
5. Cohesion	185	6.57	2.30	0.486^**^	0.310^**^	0.216^**^	−0.495^**^	——				
6. Expressiveness	185	5.15	1.97	0.420^**^	0.349^**^	0.222^**^	−0.171^*^	0.592^**^	——			
7. Gender	185	1.49	0.56	−0.068	−0.155^*^	−0.097	0.159^*^	−0.003	−0.075	——		
8. Age	185	20.59	1.52	−0.018	−0.101	−0.083	−0.125	0.065	0.038	0.057	——	
9. Only child	184	1.58	0.49	−0.022	−0.011	−0.06	−0.091	0.102	−0.073	−0.076	0.585^**^	——
10. City tier	185	2.28	1.38	−0.016	0.074	0.014	−0.158^*^	0.099	−0.033	−0.043	−0.011	0.014

For gender, “male” coded as 1 and “female” coded as 2; for only child, “only child” coded as 1 and “multi-child” coded as 2; for marital status, “divorced” coded as 1, “widowed” coded as 2 and “married” coded as 3; for city tier, “first tier city” coded as 1, “second tier city” coded as 2, “third tier city” coded as 3, “other tier city” coded as 4.

* Correlation is significant at the 0.05 level (two-tailed).

** Correlation is significant at the 0.01 level (two-tailed).

### Correlation analysis by gender, age, class year, city tier, and only-child status

Pearson correlation coefficients were calculated separately for men and women participants to examine the relationships between PPE, self-efficacy, and resilience. The sample sizes of other genders were too small to yield reliable results, so those participants (*n* = 6) were excluded from the analysis. There were significant differences between men and women self-efficacy scores, *t*(177) = 2.11, *p* = 0.037 and resilience scores, *t*(177) = 2.37, *p* = 0.019, with men students’ scores (*M* = 26.65) and women students’ scores (*M* = 24.95). There was no significant difference between men (*M* = 43.42) and women (*M* = 43.98) PPE scores. However, the positive correlation between PPE and self-efficacy among women, *r*(77) = 0.37, *p* < 0.001 was stronger than that among men, *r*(98) = 0.20, *p* < 0.05. Similarly, the positive correlation between PPE and resilience among women, *r*(77) = 0.42, *p* < 0.001, was greater than that among men, *r*(98) = 0.36, *p* < 0.01.

To compare self-efficacy scores between only-child families and multi-child families, an independent sample *t*-test was conducted. 77 participants are from only-child families, and 107 participants are from multi-child families. One participant did not answer the question. The results revealed a significant difference, *t*(182) = 2.03, *p* = 0.044 between only-child families (*M* = 26.80) and multi-child families (*M* = 25.16). The positive correlation between PPE and resilience among multi-child families, *r*(107) = 0.46, *p* < 0.001, was stronger than resilience among only-child families, *r*(75) = 0.30, *p* < 0.01. There is a positive correlation between PPE and self-efficacy among multi-child families, *r*(105) = 0.38, *p* < 0.001, while there is no correlation between PPE and self-efficacy among only-child families.

A total of 91 participants are from the first city tier group; 18 participants are from the second city tier group; 10 participants are from the third city tier group, and 66 participants are from the fourth city tier group. There was a positive correlation between PPE and self-efficacy in the first city tier group, *r*(89) = 0.27, *p* < 0.001, and in the fourth city tier group, *r*(64) = 0.31, *p* < 0.01. A medium positive correlation was found between PPE and resilience in the first city tier group, *r*(89) = 0.34, *p* < 0.01, and in the fourth city tier group, *r*(64) = 0.43, *p* < 0.001. There was a significance difference between city tiers in self-efficacy, *F*(3, 181) = 2.72, *p* = 0.046. No significant differences in self-efficacy and resilience were found between the second city tier group and the third city tier group using Tukey’s *post hoc* analysis (see Table 2).

### Main analysis

Two linear regression analyses were conducted to examine the relationship between PPE and self-efficacy and resilience, respectively. The results showed that PPE positively predicted self-efficacy (*β* = 0.18, *p* < 0.001, 95% confidence interval [CI]: [0.087, 0.280]), and *F*(1, 183) = 13.94, *p* < 0.001. Similarly, PPE positively predicted resilience (*β* = 0.30, *p* < 0.001, 95% CI: [0.191, 0.403]), and *F*(1, 183) = 30.39, *p* < 0.001.

### Moderation analyses

Hierarchical multiple regression was used to assess whether family environment indicators (Conflict, Cohesion, and Expressiveness) moderated associations between parental encouragement and (a) self-efficacy and (b) resilience. Following a factor-specific moderation approach, six separate moderation models were estimated (three moderators × two outcomes). Centered scores were used to reduce multicollinearity by avoiding interaction terms that are highly correlated with the main effects. Interaction terms were computed as the products of centered variables. For each model, Step 1 included PPE and the principal moderator, and Step 2 added the corresponding interaction term (PPE × moderator). The incremental contribution of the interaction was evaluated using Δ*R*^2^ and its significance, alongside the interaction coefficient and its 95% confidence interval.

Since six interaction effects were assessed, a Bonferroni correction was applied to interaction terms (*α*_adj = 0.05/6 = 0.0083). For self-efficacy, none of the interaction terms were statistically significant (PPE × Conflict, PPE × Cohesion, PPE × Expressiveness), and Δ*R*^2^ values with each interaction were small. For resilience, the PPE × Conflict interaction was statistically significant and remained significant under the Bonferroni-adjusted threshold (*B* = −0.06, SE = 0.022, *β* = −0.20, *t* = −2.84, *p* = 0.005, 95% CI: [−0.107, −0.019]). The negative interaction coefficient indicates that higher Family Conflict lessens the positive association between PPE and resilience. The PPE × Cohesion and PPE × Expressiveness interactions were not significant under Bonferroni correction (see Tables 3, 4).

**Table 3:** Moderation table for self-efficacy.

Self-efficacy												
Predictor	B	SE	*β*	*t*	*p*	95% CI	*R*^2^ Step 1	*R*^2^ Step 2	Δ*R*^2^	*p* (Δ*R*^2^)	Bonferroni *p*_adj	Significance (Bonferroni *α*_adj = 0.0083)
Model 1 (Family Conflict)												
PPE	0.2	0.055	0.29	3.605	<0.001^**^	[0.090, 0.309]	0.071	0.087				
Conflict	−0.037	0.182	−0.016	−0.201	0.841	[−0.396, 0.323]						
PPE × Conflict	−0.037	0.02	−0.133	−1.817	0.071	[−0.078, 0.003]			0.017	0.071	0.426	False
Model 2 (Family Cohesion)												
PPE	0.167	0.058	0.242	2.891	0.004^**^	[0.053, 0.280]	0.081	0.092				
Cohesion	0.319	0.194	0.136	1.644	0.102	[−0.064, 0.702]						
PPE × Cohesion	0.03	0.02	0.116	1.497	0.136	[−0.009, 0.069]			0.011	0.136	0.816	False
Model 3 (Family Expressiveness)												
PPE	0.148	0.055	0.214	2.699	0.008^**^	[0.040, 0.255]	0.086	0.086				
Expressiveness	0.378	0.217	0.138	1.746	0.082	[−0.049, 0.806]						
PPE × Expressiveness	0.009	0.025	0.026	0.347	0.729	[−0.040, 0.058]			0.001	0.729	1	False

** *p*-value is significant at the 0.01 level (2-tailed).

**Table 4:** Moderation table for resilience.

Resilience												
Predictor	B	SE	*β*	*t*	*p*	95% CI	*R*^2^ Step 1	*R*^2^ Step 2	Δ*R*^2^	*p* (Δ*R*^2^)	Bonferroni *p*_adj	Significance (Bonferroni *α*_adj = 0.0083)
Model 1 (Family Conflict)												
PPE	0.34	0.06	0.432	5.677	<0.001^**^	[0.222, 0.458]	0.144	0.18				
Conflict	0.055	0.197	0.021	0.28	0.780	[−0.334, 0.444]						
PPE × Conflict	−0.063	0.022	−0.197	−2.84	0.005^**^	[−0.107, −0.019]			0.037	0.005^**^	0.03	True
Model 2 (Family Cohesion)												
PPE	0.258	0.063	0.328	4.122	<0.001^**^	[0.135, 0.382]	0.163	0.175				
Cohesion	0.508	0.211	0.189	2.409	0.017^*^	[0.092, 0.925]						
PPE × Cohesion	0.035	0.022	0.12	1.628	0.105	[−0.007, 0.078]			0.012	0.105	0.63	False
Model 3 (Family Expressiveness)												
PPE	0.222	0.059	0.282	3.771	<0.001^**^	[0.106, 0.338]	0.187	0.187				
Expressiveness	0.731	0.233	0.233	3.132	0.002^**^	[0.270, 1.191]						
PPE × Expressiveness	0.005	0.027	0.013	0.18	0.857	[−0.048, 0.058]			0	0.857	1	False

* *p*-value is significant at the 0.05 level (2-tailed).

** *p*-value is significant at the 0.01 level (2-tailed).

## Discussion

Given the limited research on positive parenting in the Chinese context, the present study examined the role of PPE in self-efficacy and resilience among Chinese college students. The results supported our hypotheses that there is a significant positive relationship between PPE, self-efficacy, and resilience. These findings are consistent with the existing literature, which indicates that parental encouragement fosters a sense of competence and adaptive coping in their children (Wang et al., 2007). These findings are not only consistent with what is suggested by Ecological Systems Theory that the interaction between parents and children in the microsystem could be generalized to other systems and produced positive psychological outcomes (Bronfenbrenner, 1979) but also reflect broader sociocultural transformations in Chinese parenting practices. It is a great trend that contemporary Chinese parenting practices are increasingly adopting a dual emphasis on *guan* and emotionally supportive strategies to cultivate adolescents’ psychological flexibility, including self-efficacy and resilience (Zhang and Thomas, 1994).

Gender role expectations may shape adolescents’ socialization, and how parental encouragement is communicated and interpreted. Consistent with the idea of genderized social norms, recent Chinese studies have documented that family functioning and resilience processes can differ by gender (Zhang et al., 2024) and that parenting dynamics under educational pressure may operate differently for boys vs. girls (Lu et al., 2025). Supportive relationships (including parental relationships and peer friendships) may function as protective factors for girls’ adjustment (Liu, 2021). Importantly, however, these patterns should not be assumed to be universal or biologically inherent; rather, they are best understood as culturally embedded socialization tendencies that may vary across families, regions, and historical cohorts.

In the present study, women reported stronger positive associations between PPE and both self-efficacy and resilience than men. Future research should examine whether culturally embedded messages that combine filial expectations with encouragement to foster autonomy are associated with self-efficacy and resilience among girls and boys, using designs that can directly assess these socialization processes and their impacts.

Among only children, the non-significant correlation between PPE and self-efficacy a ceiling effect. Intensive, undivided parental investment prepares a child for high self-efficacy; therefore, additional PPE offers diminishing returns. Furthermore, without sibling benchmarks, messages from PPE may be viewed less as supportive affirmation but more as a filial mandate to fulfill Confucian role obligations (Shek and Sun, 2013). In multi-child families, where parental attention from parents and resources are shared, parental encouragement becomes an important measure of individual worth, thereby elevating both self-efficacy and resilience. The stronger correlation between PPE and resilience for multi-child families suggests that PPE buffers intra-family competition and limited parental availability, fostering adaptive coping (Masten, 2001). In multi-child families, when their children enter college, they obtain greater incremental gains from PPE and self-efficacy when compared to only-child children, who come into college with a perceived higher baseline of self-efficacy, which challenges the “little-emperor” phenomenon and highlights the need to explore supportive strategies for modern Chinese family structures (Tang et al., 2025; Ye et al., 2025).

City tier may be a useful contextual indicator when describing heterogeneity in the associations among perceived parental encouragement (PPE), self-efficacy, and resilience. In the present study, self-efficacy and resilience showed a positive correlation among participants from the first-tier cities. One plausible interpretation is that adolescents in higher-tier contexts may have greater exposure to institutional and familial supports that facilitate autonomy development and coping resources, thereby enhancing self-efficacy and resilience. For example, parental education and related forms of capital may be more accessible in some higher-tier contexts. They may shape parents’ capacity to encourage adolescents’ academic and career planning and to provide psychological guidance (Tan and Fang, 2023). At the same time, correlations observed in the fourth-tier contexts should not be framed as evidence of “resilience-through-adversity.” The present study cannot determine whether PPE functions as a compensatory buffer for contextual disadvantage, nor can it identify which specific contextual factors (e.g., school quality, family socioeconomic status, migration history) influence the observed tier-specific patterns. Future work should incorporate these contextual indicators to directly evaluate these relationships.

Our results indicated that higher levels of Family Conflict reduced the positive effect of PPE on resilience. This finding suggests the significance of family harmony in Chinese people’s lives. There is a traditional and well-accepted belief, “家和万事兴,” which means “harmony in the family leads to prosperity in all things.” This belief emphasizes the importance of maintaining harmony and peace within the family, consistent with the research finding that a harmonious home serves as the foundation for success and happiness in all aspects of life (Lam et al., 2012). Maintaining peaceful and cohesive family relationships is highly valued and is believed to be a necessary condition for children’s psychological wellbeing in Chinese culture (Xu et al., 2013). Harmonious family dynamics support the realization of advantages of supportive parenting practices, while Family Conflicts may undermine the environment needed for encouragement to effectively bolster resilience (Cheung et al., 2020).

Contrary to our expectations, Family Expressiveness and Family Cohesion did not moderate the relationship between PPE, self-efficacy, and resilience. This contradiction might be due to measurement bias, as Family Expressiveness and Family Cohesion measures inherited Western cultural values and family structures, which may not directly capture the Expressiveness of Chinese family dynamics. For example, “family members often keep their feelings to themselves” and “we said anything we wanted around home.” The fact that Family Cohesion and Family Expressiveness were positively correlated with self-efficacy and resilience suggested the importance of family harmony for children’s psychological wellbeing (Shen, 2011). Its moderating role needs further investigation.

## Limitations

This study’s findings should be interpreted in light of certain limitations. Participants were recruited using online social media postings and thus constitute a non-probability convenience sample. As a result, estimates may reflect self-selection (e.g., differential likelihood of survey participation among students who encountered the recruitment posts), and generalizability to the broader population of Chinese college students may be limited. Second, the sample, while diverse in geographic representation, may not fully capture the heterogeneity of Chinese adolescents, as it included only college students, not trade school students or non-degree-seeking individuals, potentially limiting generalizability, as they may differ in SES and family environment. Additionally, due to the small sample size, we did not have enough participants in the second and third city tiers, which prevented us from fully exploring potential differences across all city tiers. Finally, cultural nuances specific to China may not apply to Chinese populations in other sociocultural contexts.

## Implications and future research directions

The findings of this study suggest several practical implications. The results indicate that higher perceived parental encouragement is generally associated with greater self-efficacy and resilience within a broader family environment. In particular, Family Conflict appears to be a clinically relevant contextual factor; therefore, encouragement-focused conceptualizations and interventions may be most effective when they simultaneously address the relational environments in which parental messages are delivered. Our results support intervention programs similar to PALS (Landry et al., 2008) and the Family Check-Up (Hentges et al., 2020). These programs directly target parental communication, Conflict management, and emotional safety.

In clinical practice, clinicians working with Chinese adolescents or Chinese college students may incorporate a brief family-informed module during treatment planning, which involves screening for Family Conflict and determining the need for Conflict-reduction skills. For adolescents’ families characterized by high Conflict, clinicians should focus on stabilizing the relational climate by increasing emotional safety and reducing escalation. Establishing agreed-upon communication rules after difficult conversations (e.g., avoiding insults and comparisons), using brief time-out and return-to-topic strategies when emotions intensify, and teaching repair routines after disagreements (e.g., acknowledging harm and apologizing for tone) can help parents enhance the credibility of subsequent supportive messages conveyed to adolescents.

Once emotional safety is ensured, parental encouragement skills that are likely to strengthen adolescents’ self-efficacy and resilience can be applied. Using specific, process-focused encouragement that highlights efforts, offering structured choices within culturally valued expectations, expressing confidence in adolescents’ capacity, and using collaborative problem-solving rather than lecturing would be plausible approaches for clinicians to help parents practice within their family’s structure.

In school and university mental health services, where parent involvement is not always feasible or appropriate, the findings still offer actionable guidance for student-centered care (Kamal Ariffin et al., 2022; Thériault et al., 2021). School and university mental-health services may offer individual or group programs that (a) strengthen self-efficacy through mastery planning, goal scaffolding, and coping rehearsal; (b) help students clarify boundaries and select safer communication strategies with parents (e.g., timing conversations, limiting triggering topics, and using brief scripts). When clinically indicated and safe, optional parent outreach (such as brief webinars or handouts) can provide effective guidance on parental encouragement and Conflict de-escalation, while preserving students’ independence and autonomy. This flexible approach is important because family engagement may be culturally sensitive and difficult for some students to practice.

Building on these findings, future studies could examine longitudinal trajectories to identify causal pathways among PPE, self-efficacy, and resilience. Investigating the role of additional family dynamics, such as parental control or warmth, may offer deeper insights into familial influences. Furthermore, examining these relationships across diverse cultural settings through broader recruitment channels could strengthen the universality or specificity of these correlations. Intervention-based research assessing the efficacy of programs aimed at enhancing perceived parental encouragement and reducing Family Conflict would also be valuable. Given the findings of this study and the current gap in the literature, there is a clear need to develop or enhance a scale specifically designed to measure PPE, as this would allow for a more precise assessment of this construct and its impact on psychological outcomes like self-efficacy and resilience, as well as ensuring it is culturally adaptable and psychometrically sound. Future research should include participants from diverse cultural backgrounds and regions to validate these findings and provide a more comprehensive understanding of how PPE operates across diverse cultural contexts.

## Conclusion

This study shows the importance of PPE for the psychological wellbeing of Chinese college students. The findings of this study suggest that PPE matters even as adolescents become more independent and face increasing academic and developmental pressures. At the same time, the findings show that the impact of PPE depends on the broader family environment. PPE does not operate in isolation. Ongoing tension and Conflict may make it harder for adolescents to fully internalize their parents’ supportive messages and enhance their own psychological wellbeing. Therefore, PPE appears to be most helpful when it occurs within a relatively harmonious family setting.

Given the study’s cross-sectional design and convenience sampling, future research should explore longitudinal and multi-site recruitment strategies, which may provide a more nuanced understanding of how perceived parental encouragement operates within Chinese families.

Overall, this study adds to research on positive parenting in China by demonstrating that PPE is linked to psychological wellbeing among college students, while clarifying that Conflict in the family can constrain these associations. These findings show the value of considering both supportive parenting experiences and the broader family when examining developmental adaptation in Chinese adolescents.

## Data Availability

The raw data supporting the conclusions of this article will be made available by the authors, without undue reservation.
